# Correction: Youth friendly sexual and reproductive health service utilization among high and preparatory school students in Debre Tabor town, Northwest Ethiopia: A cross sectional study

**DOI:** 10.1371/journal.pone.0242850

**Published:** 2020-11-18

**Authors:** Amare Simegn, Telake Azale, Abebaw Addis, Mulugeta Dile, Yitayal Ayalew, Binyam Minuye

The sixth author's name is spelled incorrectly. The correct name is: Binyam Minuye.
