# Supplementary material for: Youth friendly sexual and reproductive health service utilization among high and preparatory school students in Debre Tabor town, Northwest Ethiopia: A cross sectional study
Source: PLoS One. 2020 Sep 30;15(9):e0240033. doi: 10.1371/journal.pone.0240033 (PMC7526918; doi:10.1371/journal.pone.0240033)
Supplement: Attachment — Submitted filename: Summary of revie-PONE-D-19-33641.docx [file pone.0240033.s002.docx]

**Summary of the article**

The manuscript "Youth friendly health service utilization among high and preparatory school students in Debre Tabor Town, Northwest Ethiopia" tries to assess utilization and factors affecting utilization among youths. The study may have minimal contribution to the context of the study area if the following major and minor comments are addressed by the:

1. **General comment**

- First of all, the issue is overlooked in various parts of the country. Some of these includes Jimma (South West Ethiopia), Harar (East ethiopia) , Anchar district (East ethiopia ) , Ethiopia (Meta analysis ), Badwacho (Southern Ethiopia), Wolaita ( Southern Ethiopia), Hossana ( Southern Ethiopia), Asela ( South East Ethiopia), Bahir Dar ( North Ethioia ), Tigray ( North Ethiopia), Debre Birhan ( North Ethiopia), Nekemte ( West Ethiopia), Awabel district ( North west Ethiopia), Metekel Zone ( North West Ethiopia), Goba town ( South East Ethiopia), Dejene district ( North Ethiopia), Debre Markos (North Ethiopia), Woreta Town ( North West Ethiopia), Kachabira District ( Southern Ethiopia), Meachkal Distric ( North Wets Ethiopia), and Mecha district ( North west Ethiopia). If we commutate all findings, the utilization of “youth friendly sexual and reproductive health service” is very low. So what ? Does this study come with a new perspective ? Yes, we know the contexts particularly for these subgroup populations are different in different areas due to individual level factors, structural factors, sociodemographic factors, and so on. Majority of the studies including the current study are done in towns and all are from youth/adolescent perspectives of the services.
- The justification “the status of these segments of population in using sexual and reproductive health services is not clearly known in the country” in the abstract section is not palatable and therefore, need another peculiar justification.
- There are issues related to the journal’s guideline like reference writing consistency

2. **Abstract**

- See the track change comments

3. Methods section

- See the track change comments

4. **Results Section**

- See the track change comments

5. **Reference and other issues**

- See the track change comments
