# Supplementary material for: Youth friendly sexual and reproductive health service utilization among high and preparatory school students in Debre Tabor town, Northwest Ethiopia: A cross sectional study
Source: PLoS One. 2020 Sep 30;15(9):e0240033. doi: 10.1371/journal.pone.0240033 (PMC7526918; doi:10.1371/journal.pone.0240033)
Supplement: Attachment — Submitted filename: comment paper plos one.docx [file pone.0240033.s004.docx]

Comments

This is important topic which highlighted issues among youth.

I would like to provide my comments to improve this paper further.

Background

1. Generally you have mentioned the important point regarding issue in this study, however literature seems very brief. I would suggest to add on more
2. Paragraph 3… ‘They lack RH information, knowledge and access………service for RH’ is not clear. Maybe you could restructure the sentences
3. The acronym used in highlighting the issues not consistent as you used for SRH, RH and ARH. My suggestion is used consistency term and acronym that best applied to this study. Using full terminology rather than acronym is much better.
4. In Paragraph 6, acronym for YFS means what?

Methodology

1. How many population in this town and may also include how big is the town.
2. Please elaborate detail on sample size calculation; how you get 696 students participated in this study. How you did simple random sampling? How many school involved?
3. Study instrument is not clear and need further elaboration, eg what questionnaire you used, how many questions, how it is developed and does it is validated
4. In writing the variables I suggest to use proper sentences. Also maybe it is very nice to put point or numbers in writing the operational definition or maybe just highlighted the variable /operational definition related to your result and discussion rather put it all as listed
5. In ethical consideration, since your sample /participants are between age 15 to 24 years old, the ethical address should properly highlighted. Maybe you should mention at what age respondent can give consent and for those under children age (less than 18 years) how consent form was taken and should also mention others ethical issues as this topic or question that you asked give impact to subject.

Result & Discussion

1. Is well presented however should report detail on response rate in this study? Maybe report the reason why they rejected to participate?
2. In the discussion , paragraph 2 …(M:F, 50.9:49.1)….what does it means? Is it references?

However, maybe this study only represented youth who are in the education system since your population is not cover among those who did not enter school. This should be discuss in the limitation and maybe suggestion to improve in research.

Conclusion & Limitation

Should explain detail
