# Supplementary material for: Youth friendly sexual and reproductive health service utilization among high and preparatory school students in Debre Tabor town, Northwest Ethiopia: A cross sectional study
Source: PLoS One. 2020 Sep 30;15(9):e0240033. doi: 10.1371/journal.pone.0240033 (PMC7526918; doi:10.1371/journal.pone.0240033)
Supplement: Attachment — Submitted filename: PLOS 1_Reviewer comments_ Manuscript YFHSU 240220.docx [file pone.0240033.s005.docx]

**Reviewer attachment for Manuscript Number PONE-D-19-33641 - Youth friendly health service utilization among high and preparatory school students in Debre Tabor Town, Northwest Ethiopia**

**Background**

Page 1, line 1: Grammar error - "Reproductive health is 'a'..."

Page 1, line 1-2: Definition of ‘reproductive health’ – this is a direct quote. Please include quotation marks.

Page 1, line 4: "Every year an estimated 1.7 million youths lose their lives prematurely". Were you referring SRH problems? Please specify.

Page 1, para 2: "Individuals in the age group of 15-24 years are characterized by significant physiological, psychological and social changes and making up about 20% of the world’s population, 85% lived in developing countries" - This sentence is too long and confusing. Suggest to rephrase the sentence.

Page 1, para 3: adolescent reproductive health (RH) - please be consistent with the abbreviation used. ARH or RH?

**Methods**

Page 2, **Study period, Setting, and Population**, para 1 - The study duration is not the same as stated in the abstract.

Why Debre Tabor town was chosen as the study setting? Please justify.

Page 3, para 1 – “There are five privately owned kindergartens, 12 government and private primary schools, four government senior secondary schools (9-10), two preparatory schools, one Teachers vocational educational training, two public and one private college, and one University”- Are all these schools and colleges located in Debre Tabor town? Please be clear.

Page 3, para 1: "In the town, there were 4152 (2242 female and 1910 male) high schools and 2955 (1459 male and 1496 female) preparatory students" - Are high schools the same as secondary schools? This needs to be clarified from the start.

Page 3, para 2 - "Night and extension students were excluded" - why were they excluded?

Page 3, para 2 - "A total of 696 students were participated..." - Grammar error

Page 3, para 2 - "Samples were drown..." - drawn

How did the author do simple random sampling? Was random table or computer used to generate the samples?

Why are there two subtitles? - **Data Collection Methods & Data Collection Procedure**. Suggest 'Data Collection'.

Page 3, last paragraph - "Data were collected by using structured close ended self-administered questionnaire. Data were collected by six nurses during working hours’ and supervised by three public..." - 'data were collected' used in two consecutive sentences.

Page 3, last paragraph: "Data were collected by six nurses during working hours..." - is it necessary to state the 'working hours'?

Page 3, last paragraph: The questioner was prepared in English and translated in “Amharic” for data collection and retranslated into “English” - spelling error questionnaire and you may want to use the term 'forward and backward translation' when describing the translation process.

Page 4: Dependent variable - Youth friendly health service utilization - there is no description of YFHSU. How was it measured?

Family characteristics - how was it measured?

Is the term definition for 'Reproductive health service utilization' the same as 'Youth friendly health service utilization'? Please be consistent.

Page 4: Were the variables defined under 'Operational Definitions' adopted from a validated questionnaire or did the author developed their own questionnaire? This needs to be clearly stated in the manuscript.

Page 4: Youth-communication/discussion: “Youth report having talked to anyone else about one or more SRH services were categorized as having a communication/discussion about the services” - please specify the people whom they had conversations with e.g. family, teachers etc.

Page 5, Youth preferences: 'Preference of youth in relation to health care workers' and 'time focusing SRH services' - were these two sub-variables separated in the questionnaire?

How was sample size calculated? This needs to be described.

Ethical considerations - was parental consent obtained? In some countries e.g. Malaysia, those less than 18 years old are considered minors and parental consent is required.

Ethical considerations - The purpose of the study was explained to the study participants, informed written consent and assent were secured and confidentiality of the information was censured - why confidentiality was ‘censured’? Did you mean to say assured?

**Results**

Page 6, 'sources of information' - please specify. Was it sources of info on SRH?

Page 7, RHSU: A total of 690 school youths were participated. – grammar error and avoid repetition.

**Discussion**

Page 8: “The possible explanations for the difference of the study in Harar could be justified as a higher proportion of married respondents (16.3%) in Harar may result in a higher proportion of service utilization”. - To include in-text citation.

Page 8-9: “This might be due to differences in the availability and accessibility of youth friendly health facilities, youth centers, educational status, socioeconomic status, type of residence, transportation and culture”. - To include in-text citation.

Page 9: This might be due to cultural influences in the study area in which females have been still not allowed to go to health facilities for reproductive health services. It might also be due to the fact that the proportion of male and female participants in our study was different from Badewacho woreda’s (M: F, 50.9:49.1) - Provide evidence for these two points.

Page 9-10: This might be due to the fact that many of reproductive health service components (contraception) might be used when youths have perceived reproductive health risks related to sexual intercourse. - Is this your assumption? Provide evidence.

Page 10: This might probably be due to the reason that students spent their time at school during the regular health institutions’ working hours and the institution may not be possibly functional in the weekend at which the students are relatively free. Besides, since the society declares the students as they are too young to go to the health institution due to some cultural influences and visiting health institutions for particular SRH services might be thought as shameful. - Provide evidence

Page 10: Besides, the students may also perceive that long queue may let them for unnecessary exposure to the peoples who might be around the health institution and this exposure might leave frustration related to their privacy and confidentiality. Hence, the situation might be exacerbated if they think that the location of the health institution is inconvenient for them. - Provide evidence.

Page 11: Limitations - authors should discuss both the strengths and limitations in the discussion section.

Page 11: Difficult to determine the direction of causality. Factors from the service providers’ perspective, structural barriers as well as the supply was missed. - These limitations should have been discussed using proper sentences and elaborated further. Authors should also include actions to overcome those challenges.

Page 11: Factors from the service providers’ perspective, structural barriers as well as the supply was missed. - Why was it missed? This should not have happened if a comprehensive literature review was done.

Table 1: Why there were more adolescents from age group 15-19 years old?

Figure: Should be labelled as Figure 1.
