# Supplementary material for: Youth friendly sexual and reproductive health service utilization among high and preparatory school students in Debre Tabor town, Northwest Ethiopia: A cross sectional study
Source: PLoS One. 2020 Sep 30;15(9):e0240033. doi: 10.1371/journal.pone.0240033 (PMC7526918; doi:10.1371/journal.pone.0240033)
Supplement: Attachment — Submitted filename: Response to reviewers.docx [file pone.0240033.s006.docx]

Dear Editors and reviewer;

We sincerely appreciate the valuable comments and suggestions from the reviewers and editors. The thorough review helped immensely in the shaping of the manuscript. The suggestions and comments have been closely followed and revisions have been made accordingly. The following are the questions extracted from the reviewers’ comments along with our summarized responses. Thank you very much for your constrictive comments. I tried to inculcate your comments and questions as described below. The changes will be attached with.

Reviewer #1: Summary of the article
the manuscript "Youth friendly health service utilization among high and preparatory school students in Debre Tabor Town, Northwest Ethiopia" tries to assess utilization and factors affecting utilization among youths. The study may have minimal contribution to the context of the study area if the following major and minor comments are addressed by the:
1. General comment
• First of all, the issue is overlooked in various parts of the country. Some of these includes Jimma (South West Ethiopia), Harar (East ethiopia) , Anchar district (East ethiopia ) , Ethiopia (Meta-analysis ), Badwacho (Southern Ethiopia), Wolaita ( Southern Ethiopia), Hossana ( Southern Ethiopia), Asela ( South East Ethiopia), Bahir Dar ( North Ethioia ), Tigray ( North Ethiopia), Debre Birhan ( North Ethiopia), Nekemte ( West Ethiopia), Awabel district ( North west Ethiopia), Metekel Zone ( North West Ethiopia), Goba town ( South East Ethiopia), Dejene district ( North Ethiopia), Debre Markos (North Ethiopia), Woreta Town ( North West Ethiopia), Kachabira District ( Southern Ethiopia), Meachkal Distric ( North Wets Ethiopia), and Mecha district ( North west Ethiopia). If we commutate all findings, the utilization of “youth friendly sexual and reproductive health service” is very low. So what? Does this study come with a new perspective? Yes, we know the contexts particularly for these subgroup populations are different in different areas due to individual level factors, structural factors, Sociodemographic factors, and so on. Majority of the studies including the current study are done in towns and all are from youth/adolescent perspectives of the services.
• The justification “the status of these segments of population in using sexual and reproductive health services is not clearly known in the country” in the abstract section is not palatable and therefore, need another peculiar justification.
• There are issues related to the journal’s guideline like reference writing consistency

Authors Response: Accepted and corrected

2. Abstract
• Authors Response: Accepted and corrected
3. Methods section
Authors Response: The sample size was determined by using Epi info 7 statistical software by single population proportion based on following assumption

1. Estimated proportion (p) taken from previous study done at hadiya zone on the utilization of reproductive health services among youth (**29.4%**)
2. Margin of error d= 5%
3. Confidence level of 95% is assumed(Z^2^_α/2=1.96)_

n = Z^2^_α/2_(P (1-P)/d^2^ +10%

Z= is the standard normal value corresponding to the desired level of confidence

d=margin of errors

p= is the estimated proportion of an attribute that is present in the population

n=	Z^2^_α/2_(P (1-P)/d^2^

= (1.96)^2^(0.29) (0.71)

(0.05)^2^

n=316, multiplying by nonresponse rate (10%) and design effect of 2

Final sample size (nf) = [316+ (316*10%)] * 2	= **696**

Youth friendly health service utilization(Yes, No) was measured as defined on operational definition ; youth practice of utilizing reproductive health services(family planning, sexually transmitted infections, diagnosis and treatment for reproductive health issues, voluntary counselling and testing, antenatal care, abortion care, post abortion care, condom use and general health information/counselling in the past one year

It does not include diagnosis and treatment services other than reproductive health issue. Family planning was frequently facing youths.

**Sampling technique**

Multi stage cluster sampling method was used to select the study participants. There were 4 high schools and 2 preparatory schools in Debre Tabor town. The number of respondents in the two schools (secondary and preparatory) was determined using probability proportionate to size allocation method. Then, 24 sections (6 sections for each high school) from a total of 81 high school sections were randomly selected. Similarly, 19 sections (12 sections from pre-1 and 7 sections from pre-2) were randomly selected from total of 64 preparatory sections. Again the students from these eligible sections were randomly selected using the list of student’s ID number as a sampling frame. Finally, the data was collected from 400 high school students and 290 preparatory students which result in the total of 690 respondents.

**Debre Tabor regular secondary and preparatory schools**

**2- Preparatory schools (A total of 2935 Stu =64 sections**

**4-High schools (A total of 4152 students = 81 sections)**

**Simple random sampling method was used to select the required sections**

**Simplsioo**

**19 – Sections (874 Students)**

**24 – Sections (1224 Students)**

| **12 sec**  **Pre-1** | **7 sec**  **Pre-2** |
| --- | --- |

| **6 sec**  **HS-1** | **6 sec**  **HS-2** | **6 sec**  **HS-3** | **6 sec**  **HS-4** |
| --- | --- | --- | --- |

**Simple random sampling method was used to select the study participants**

**99**

**189**

**102**

**102**

**102**

**102**

**Keys**

**HS- High school**

**Final sample size =696**

**Pre- Preparatory**

**Sec-Section**

Figure 2: Schematic presentation of sampling procedures

Sexual activity: Refers to the involvement in a penetrative heterosexual intercourse within the last 12 months.

Author response: We use penetrative –to decrease recall bias ;heterosexual –we perceive homosexual is not such practiced in the study area.

Because penetrative

Awareness about SRH Services: Those knowing the availability of one or more SRH service everywhere(health institutions ,shop, drug stores ,school, market…) were reported as were reported as having awareness about the services.

How consistency of the entered data was performed? What type of consistency measures you used? Was

There the level at the acceptable range? What was it?
Consistency of data was done (1^st^ sorting the data then compare each variables) .
4. Results Section
• Author response: the comments were accepted and corrected.
5. Reference and other issues

Author response: the comments were accepted and corrected based on Plos guideline, but there is some limitation on reference section due to insufficient internet connection in our country due to COVID-19 pandemic.

This assumption mostly works for comparative designs	and has critical predictor’s nature. Your design is cross-sectional. In most cases, the cut of point of P-value is 5% ? Therefore, why is it important for you to use the cut of points? And there are comparisons in your finding with other study who were not using this assumptions. How did you see this one? Is it a just? In a bivariate logistic regression	was considered to get into your multivariate analysis while your cut of point in the case of multivariate analysis is P-value<0.05 . Why? What did you do for those who said that they did not use the service?

Author response: Multivariable and multivariate are different, since there is one dependent variable which is dichotomy, we used binary logistic regression model .The appropriate model for this study is binary logistic regression model. So we can say multivariable analysis.

Scholars suggests that variable having P-Values <0.25 in bivariable logistics regression entered into multivariable analysis. And in multivariable analysis we used the cutoff point of P-value<0.05

We disseminate the finding to schools and Woreda health office, rather it is difficult identifying individuals specifically who utilized /not utilized. Based on the finding and recommendation the responsible organization will goes to action.

Why you used for your proportion ? Robust assumption? What would have been presented only the proportion?

We can put proportion without CI, but we put to make it easy for reader and CI is the base for discussion with other literatures.

Reviewer#2: Comments
Background
1. Generally you have mentioned the important point regarding issue in this study, however literature seems very brief. I would suggest to add on more
2. Paragraph 3… ‘They lack RH information, knowledge and access………service for RH’ is not clear. Maybe you could restructure the sentences
3. The acronym used in highlighting the issues not consistent as you used for SRH, RH and ARH. My suggestion is used consistency term and acronym that best applied to this study. Using full terminology rather than acronym is much better.
4. In Paragraph 6, acronym for YFS means what?

Authors response: corrected based on the comments
Methodology
1. How many population in this town and may also include how big is the town.

Author’s response Debretabor town has a total population of 55,157(FDRE, 2008). Regarding sample size calculation –addressed in reviewer 1
2. Please elaborate detail on sample size calculation; how you get 696 students participated in this study. How you did simple random sampling? How many school involved?

Author’s response regarding sample size calculation –addressed in reviewer 1.sample size was proportionally allocated for each schools. Six schools were included in the study. We found list of students from each school, we select the participants randomly by using computer method.
3. Study instrument is not clear and need further elaboration, eg what questionnaire you used, how many questions, how it is developed and does it is validated

Author’s response a total of 37 questions which consists of socio demographic, family characteristics, respondent’s awareness and sources of information about the services, and youth preferences for time and health care workers related factors. The tool was adapted from similar previous studies.
4. In writing the variables I suggest to use proper sentences. Also maybe it is very nice to put point or numbers in writing the operational definition or maybe just highlighted the variable /operational definition related to your result and discussion rather put it all as listed

Author’s response: accepted and modified
5. In ethical consideration, since your sample /participants are between age 15 to 24 years old, the ethical address should properly highlighted. Maybe you should mention at what age respondent can give consent and for those under children age (less than 18 years) how consent form was taken and should also mention others ethical issues as this topic or question that you asked give impact to subject.

Author’s response: Based on Ethiopian constitution, peoples at age of 18 years and above can give consent. For those children age (less than 18 years) assent was taken from the guardians/parents.

Result & Discussion
1. Is well presented however should report detail on response rate in this study? Maybe report the reason why they rejected to participate?

Author’s response May be :-

1. self-administered questionnaire
2. Age of the participant
3. Ignorance

2. In the discussion, paragraph 2 …(M:F, 50.9:49.1)….what does it means? Is it references?
However, maybe this study only represented youth who are in the education system since your population is not cover among those who did not enter school. This should be discuss in the limitation and maybe suggestion to improve in research.

Author’s response Male to female ratio
Conclusion & Limitation –

Author’s response: those segment of population who did not enter school were missed
 Reviewer #3:

Overall, this is a clear, concise and well-written manuscript. The topic is timely and relevant. Below are my comments:
Background
Page 1, line 1: Grammar error - "Reproductive health is 'a'..."

Author’s response; Corrected
Page 1, line 1-2: Definition of ‘reproductive health’ – this is a direct quote. Please include quotation marks.

Author’s response: accepted and corrected
Page 1, line 4: "Every year an estimated 1.7 million youths lose their lives prematurely". Were you referring SRH problems? Please specify.

Author’s response Yes ,related to reproductive health problems
Page 1, para 2: "Individuals in the age group of 15-24 years are characterized by significant physiological, psychological and social changes and making up about 20% of the world’s population, 85% lived in developing countries" - This sentence is too long and confusing. Suggest to rephrase the sentence.

Author’s response: accepted and corrected
Page 1, para 3: adolescent reproductive health (RH) - please be consistent with the abbreviation used. ARH or RH?

Author’s response: accepted and corrected
Methods
Page 2, Study period, Setting, and Population, para 1 - The study duration is not the same as stated in the abstract.

Author’s response .Accepted and corrected, due to editorial error
Why Debre Tabor town was chosen as the study setting? Please justify.

Author’s response B/c 1. No previous study conducted in this setting,

2. There are reports by the schools and Woreda health office related to sexual and reproductive problems eg. Unintended pregnancy

3. Utilization of reproductive health is one gap identified by students during school and home visit

Page 3, para 1 – “There are five privately owned kindergartens, 12 government and private primary schools, four government senior secondary schools (9-10), two preparatory schools, one Teachers vocational educational training, two public and one private college, and one University”- Are all these schools and colleges located in Debre Tabor town? Please be clear.

Author’s response. Yes, all are found
Page 3, para 1: "In the town, there were 4152 (2242 female and 1910 male) high schools and 2955 (1459 male and 1496 female) preparatory students" - Are high schools the same as secondary schools? This needs to be clarified from the start.

Author’s response: In our context, they are similar but for consistency we use High school as a school, which entails grades 9 and 10

Page 3, para 2 - "Night and extension students were excluded" - why were they excluded?

Author’s response ;This is b/c night and extension students:-

1. more vulnerable to this sexual and reproductive issues
2. more aware of reproductive health issue

Therefore, utilization of the service is affected

Page 3, para 2 - "A total of 696 students were participated..." - Grammar error

Author’s response :Corrected as,”A total of 696 students participated”
Page 3, para 2 - "Samples were drown..." – drawn

Author’s response; Accepted
How did the author do simple random sampling? Was random table or computer used to generate the samples?

Author’s response; Using lottery method
Why are there two subtitles? - Data Collection Methods & Data Collection Procedure. Suggest 'Data Collection’. Corrected

Author’s response; accepted and corrected
Page 3, last paragraph - "Data were collected by using structured close ended self-administered questionnaire. Data were collected by six nurses during working hours’ and supervised by three public..." - 'data were collected' used in two consecutive sentences.

Author’s response .Corrected with some rearrangement
Page 3, last paragraph: "Data were collected by six nurses during working hours..." - is it necessary to state the 'working hours'?

Author’s response .working hour is omitted
Page 3, last paragraph: The questioner was prepared in English and translated in “Amharic” for data collection and retranslated into “English” - spelling error questionnaire and you may want to use the term 'forward and backward translation' when describing the translation process.

Author’s response .Corrected as, the questionnaire was prepared in English and translated to “Amharic “.’Forward and backward translation' was done

Page 4: Dependent variable - Youth friendly health service utilization - there is no description of YFHSU. How was it measured?

Author’s response .Youth friendly health service utilization(Yes, No) was measured as defined on operational definition ; youth practice of utilizing reproductive health services(family planning, sexually transmitted infections, diagnosis and treatment for reproductive health issues, voluntary counselling and testing, antenatal care, abortion care, post abortion care, condom use and general health information/counselling in the past one year
Family characteristics - how was it measured?

Author’s response Family characteristics-it includes educational status of the mother, father (unable to read and write, able to read and write, complete primary and secondary education, diploma and above). Occupational status of the mother (house wife, merchant, government employ, others), father (farmer, merchant, government employ, others), parents alive status (alive, died)

Is the term definition for 'Reproductive health service utilization' the same as 'Youth friendly health service utilization'? Please be consistent.

Author’s response .They are almost similar despite reproductive health service utilization is broader

Page 4: Were the variables defined under 'Operational Definitions' adopted from a validated questionnaire or did the author developed their own questionnaire? This needs to be clearly stated in the manuscript.

Author’s response The tool was adopted from previous research done, which are validated
Page 4: Youth-communication/discussion: “Youth report having talked to anyone else about one or more SRH services were categorized as having a communication/discussion about the services” - please specify the people whom they had conversations with e.g. family, teachers etc.

Author’s response .We accepted the comment, we did not consider. The operational definition deleted.

Page 5, Youth preferences: 'Preference of youth in relation to health care workers' and 'time focusing SRH services' - were these two sub-variables separated in the questionnaire?

Author’s response Yes, they were asked in separate way
How was sample size calculated? This needs to be described.

Author’s response Addressed above
Ethical considerations - was parental consent obtained? In some countries e.g. Malaysia, those less than 18 years old are considered minors and parental consent is required.

Author’s response The same is true in Ethiopia .We took parental consent for less than 18 years old
Ethical considerations - The purpose of the study was explained to the study participants, informed written consent and assent were secured and confidentiality of the information was censured - why confidentiality was ‘censured’? Did you mean to say assured?

Author’s response .It is to mean assured
Results
Page 6, 'sources of information' - please specify. Was it sources of info on SRH?

Author’s response Yes, source of information on sexual and reproductive services
Page 7, RHSU: A total of 690 school youths were participated. – Grammar error and avoid repetition.

Author’s response .Accepted and the sentence omitted
Discussion
Page 8: “The possible explanations for the difference of the study in Harar could be justified as a higher proportion of married respondents (16.3%) in Harar may result in a higher proportion of service utilization”. - To include in-text citation.

Author’s response .Accepted
Page 8-9: “This might be due to differences in the availability and accessibility of youth friendly health facilities, youth centers, educational status, socioeconomic status, type of residence, transportation and culture”. - To include in-text citation.

Author’s response. Accepted
Page 9: This might be due to cultural influences in the study area in which females have been still not allowed to go to health facilities for reproductive health services. It might also be due to the fact that the proportion of male and female participants in our study was different from Badewacho woreda’s (M: F, 50.9:49.1) - Provide evidence for these two points.

Author’s response .Male to female ratio in this study was 45.5:54.5

Page 9-10: This might be due to the fact that many of reproductive health service components (contraception) might be used when youths have perceived reproductive health risks related to sexual intercourse. - Is this your assumption? Provide evidence.

Author’s response .It is known that sexual and reproductive health utilization is to prevent problems related to SRH issue.

Page 10: This might probably be due to the reason that students spent their time at school during the regular health institutions’ working hours and the institution may not be possibly functional in the weekend at which the students are relatively free. Besides, since the society declares the students as they are too young to go to the health institution due to some cultural influences and visiting health institutions for particular SRH services might be thought as shameful. - Provide evidence

Author’s response:It is known that in Ethiopia grade 1-12th will not be given from weekend period.there are cultural influence on which youth utilization of sexual and reproductive issues,so they youth afraid going to utilize the service.
Page 10: Besides, the students may also perceive that long queue may let them for unnecessary exposure to the peoples who might be around the health institution and this exposure might leave frustration related to their privacy and confidentiality. Hence, the situation might be exacerbated if they think that the location of the health institution is inconvenient for them. - Provide evidence.

Author’s response. The evidence is cultural influence on utilization of reproductive service
Page 11: Limitations - authors should discuss both the strengths and limitations in the discussion section.

Author’s response; accepted
Page 11: Difficult to determine the direction of causality. Factors from the service providers’ perspective, structural barriers as well as the supply was missed. - These limitations should have been discussed using proper sentences and elaborated further. Authors should also include actions to overcome those challenges.

Author’s response: accepted and those variables were removed
Page 11: Factors from the service providers’ perspective, structural barriers as well as the supply was missed. - Why was it missed? This should not have happened if a comprehensive literature review was done.

Author’s response This is b/c our study was limited only student’s perecipective
Table 1: Why there were more adolescents from age group 15-19 years old?

Author’s response This is b/c the participants were from grade 9-12, in which most of them were in the age group of 15-19
Figure: Should be labelled as Figure 1.

Author’s response Corrected in the main document

Reviewer #4:

General comment: The manuscript needs some minor English language corrections should it go for publication. Moreover, the research has not shown any novelty from its inception to methods of undertaking.
Abstract and background
Background of the abstract and background section of the manuscript: Authors need to justify further the rationale to the study based on existing evidences. What they put as a gap for the study is not the real gap which exists in the Ethiopian context. A number of evidences have been documented with regard to reproductive health service utilization among youths either directly or indirectly. Had it been a study referring to the challenges or quality, it would have been quite informative and having had any policy implications. However, the paper can be improved if authors are able to point out any aspects of their work (methodological or variables) which can be considered as a novel contribution to the existing evidences.

Author’s response.accepted and included in the revised manuscript
Methods: The methods section is not written in detailed manner. Since plose one considers manuscripts with rigorously worked and detailed methodology, authors need to revise the methods section so that it can explicitly depict how the samples were estimated, what procedures were followed to select the samples, how the statistical analysis was made-whether appropriate considerations were made in checking for the assumptions of various analysis made to bring about the findings. Moreover, the tool used for data collection should be discussed in terms of its sources, validity and reliability.
Authors need to discuss on the variables they have considered in the analysis. The list of variables, their categorization and which have been dropped during the bivariable analysis and why should be discussed here in the methods section.

Author’s response: We think it has been shown in the table 4,to avoid redundancy we leave it
Results: Not well structured. The very critical issue is that authors stated that they have considered health system and health care provider related factors ad their “independent factors” and failed to address these factors in the results section. Therefore, something has fallen apart here.

It would be better if authors support their “operational definitions” with either a published article or book or any other original standard material.

Author’s response .Accepted and modified. Primarily we tried to consider but we missed to collect data on health system and health care provider related factors.

Discussion: The discussion is to shallow in terms of the implications of the findings for policy or programme improvements. What is the novel contribution of this research and how it is interpreted matters a lot while authors discuss their findings? In short authors need to consider a further substantiation of their “discussion “so that it can imply some thing beyond comparing and justifying for differences among findings from previous studies. For example, a number of studies reported a low utilization SRH services in the Amhara region of Ethiopia. What makes this study unique and additive to the scientific literature?

Author’s response: the contribution of the study is: - shows barriers in the study setting, baseline for implementation of youth friendly health services
Conclusion: I would suggest authors to incorporate the recommendations written with the conclusion and the limitations to the ‘discussion” section.

Author’s response. Accepted
Last-Authors did not follow the plose manuscript formatting style while drafting the manuscript.

Author’s response. Accepted and corrected per the guideline
