# Supplementary material for: Youth friendly sexual and reproductive health service utilization among high and preparatory school students in Debre Tabor town, Northwest Ethiopia: A cross sectional study
Source: PLoS One. 2020 Sep 30;15(9):e0240033. doi: 10.1371/journal.pone.0240033 (PMC7526918; doi:10.1371/journal.pone.0240033)
Supplement: Attachment — Submitted filename: Manuscript track change.docx [file pone.0240033.s008.docx]

Youth friendly health service utilization among high and preparatory school students in Debre Tabor Town, Northwest Ethiopia. A cross-sectional study design

Amare Simegn ^1^, Telake Azale ^2^, Abebaw Addis ^2 ,^ Mulugeta Dile ^1^, Yitayal Ayalew ^1^ ,Biniam Minuye ^1*^

^1^ College of Health Sciences, Debre Tabor University, Debre Tabor, Ethiopia

^2^ College of Health Sciences, University of Gondar, Gondar, Ethiopia

**Corresponding author: Biniam Minuye	Email Address: biniamminuye@yahoo.com*

AS: [amaresimegn99@gmail.com](mailto:amaresimegn99@gmail.com)

TA: [Telakea@yahoo.com](mailto:Telakea@yahoo.com)

AA: [Abebaw.addis@gmail.com](mailto:Abebaw.addis@gmail.com)

MD: [muliedile@gmail.com](mailto:muliedile@gmail.com)

YA:[ayalewyitayal@gmail.com](mailto:ayalewyitayal@gmail.com)

# Abstract

Background: Youth continue to fall victim to sexual and reproductive health problems, its consequences. Despite, reproductive health needs of youth had been supported by government and non-governmental organizations to increase access to sexual and reproductive health services by young people. All these efforts have not been felt across the Ethiopian learning institutions as is evidenced by persistent reproductive health problems and challenges of the youth including in the study setting.Therefore, this study aimed to assess the magnitude of youth-friendly health service utilization and associated factors among high school and preparatory school youths in Debre Tabor town, Northwest Ethiopia.

Methods: An institution-based cross-sectional study was conducted from March 1 to 28, 2016. The data were collected using a pre-tested and structured self-administered questionnaire. A multistage cluster sampling method was used to select the study participants. The data were entered into Epi-data version 3.1 and analyzed using SPSS version 20. Binary logistics regression was used for analysis. Odds ratio along with 95%CI was estimated to measure the strength of the association. The level of statistical significance was declared at p-value ≤0.05.

Results: Overall utilization reproductive health service was 28.8%.Being male (AOR = 1.54, 95% CI: 1.05, 2.25), prior discussion on reproductive health issues (AOR = 6.33, 95% CI: 4.22, 9.51), and previous sexual intercourse within the past one year (AOR =1.95, 95% CI: 1.10, 3.44) were significantly associated with Youth-friendly health service utilization.

Conclusions: Youth-friendly health service utilization among high school and preparatory students in Debre Tabor town was low. Ensuring gender empowerment and advocating sexual and reproductive service discussion among themselves and with others might be important in improving reproductive health utilization and health. The future researcher should address the segment of the population who does not enter school.

Keywords:Health service utilization, school, youth, Ethiopia

# Background

Reproductive health is “a state of complete physical, mental and social well-being and not merely the absence of disease or infirmity, in all matters relating to the reproductive system.” it is critical during adolescence and adulthood, and affects the health of the next generation [1]. Every year an estimated 1.7 million youths lose their lives prematurely related to reproductive health problems [2]. Due to these reasons, adolescent’s reproductive health (RH) is becoming an ever more important component of global health.

Youth are characterized by significant physiological, psychological, and social changes. This shares about 20% of the world’s population.[3, 4]. More than one-third of the Ethiopian population is aged between 15-24, most vulnerable to a range of sexual and reproductive health problems [5].

Focusing on adolescent reproductive health is both a challenge and an opportunity for health care providers. Adolescence generally is a healthy period of life, many youths are less informed on reproductive health services, less experienced, and less comfortable accessing health services for reproductive issues than adults [6-8]. Limited access and low utilization to targeted reproductive health care and services for young people contribute and exacerbate many of the RH problems [9]. These problems could be early childbearing, early sexual debut, sexually transmitted infections (STIs) including HIV/AIDS, unmet need for family planning, early marriage, and abortion[9]. Over a quarter of all pregnant youth and adolescents feel that their pregnancies are mistimed, reflecting this population’s limited access to family planning (FP) and other RH services [10]. The reasons for lower reproductive health service (RHS) utilization may include feelings of discomfort, fear of being seen by parents, and embarrassment while seeking reproductive health services [11].

Efforts have been made to address different reproductive health problems at a different level. The Ethiopian Government, along with many international non-governmental and local organizations has been supporting activities to increase access to sexual and reproductive health (RH) services for young people. This includes the scaling-up and institutionalization of Youth Friendly Services (YFS) through intensive capacity building at all levels of the health system [12, 13]. However, the effects of all these efforts have not been well understood across the Ethiopian high schools and preparatory schools as is evidenced by persistent reproductive health problems and challenges to the youth [14-16].

The utilization of reproductive health services is the key to the improvement in the quality of life of the youth. Behaviors formed and choices made during the adolescence period. Despite different studies conducted in Ethiopia, existing data didn’t fully assess the factors which affect service utilization. Therefore, this study assessed the magnitude and associated factors for Sexual and reproductive health services utilization among students in Debretabor town.

# Materials and Methods

## Study design, period, setting, and population

An institution-based cross-sectional study was employed in Debre Tabor town, Northwest Ethiopia, from March 13 to 18, 2016. Debre Tabor town is located in South Gondar Administrative Zone of the Amhara regional, state, at a distance of 667 kilometers away from Addis Ababa, the capital city of Ethiopia, 100 kilometers southeast of Gondar and 50 kilometers east of Lake Tana [17]. There are five privately owned kindergartens, 12 government and private primary schools, four government senior secondary schools (9-10), two preparatory schools, one teacher vocational educational training, two public and one private college, and one University. In the town, there were 4152 (2242 female and 1910 male) high schools and 2955 (1459 male and 1496 female) preparatory students.

All youths between the age range of 15 to 24 years who were attending their education in secondary and preparatory schools during the study period were the source population. Night and extension students were excluded. A total of 696 students participated in the study. Samples were drawn from six schools with a proportional allocation method. Finally, the simple random sampling technique was applied to get the required number of eligible students.

Sample size calculation

The sample size was determined by using Epi info 7 statistical software by single population proportion based on following assumption

1. Estimated proportion (p) taken from previous study done at hadiya zone on the utilization of reproductive health services among youth (29.4%)
2. Margin of error d= 5%
3. Confidence level of 95% is assumed(Z^2^_α/2=1.96)_

n = Z^2^_α/2_(P (1-P)/d^2^ +10%

Z= is the standard normal value corresponding to the desired level of confidence

d=margin of errors

p= is the estimated proportion of an attribute that is present in the population

n=	Z^2^_α/2_(P (1-P)/d^2^

= (1.96)^2^(0.29) (0.71)

(0.05)^2^

n=316, multiplying by nonresponse rate (10%) and design effect of 2

Final sample size (nf) = [316+ (316*10%)] * 2	= 696

Sampling technique

A multi-stage cluster sampling method was used to select the study participants. There were 4 high schools and 2 preparatory schools in Debre Tabor town. The number of respondents in the two schools (secondary and preparatory) was determined using probability proportionate to size allocation method. Then, 24 sections (6 sections for each high school) from a total of 81 high school sections were randomly selected. Similarly, 19 sections (12 sections from pre-1 and 7 sections from pre-2) were randomly selected from a total of 64 preparatory sections. Again the students from these eligible sections were randomly selected using the list of student’s ID numbers as a sampling frame. Finally, the data was collected from 400 high school students and 290 preparatory students which result in a total of 690 respondents (Figure 1).

## Data Collection

Data were collected by using a structured close-ended self-administered questionnaire. A total of 37 questions which consist of socio-demographic, family characteristics, respondent’s awareness, and sources of information about the services, health system factors, and youth preferences for time and health care workers related factors. Data were collected by six nurses and supervised by three public health professionals. The questionnaire was prepared in English and translated to “Amharic “.’ Forward and backward translation' was done. Completeness of each recording format was checked before collecting the data.

Variables

## Dependent variable

Youth-friendly health service utilization

Independent variable

Socio-demographic, family characteristics, respondent’s awareness and sources of information about the services, and youth preferences for time and health care workers related factors.

## Operational Definitions

- Reproductive health service utilization was assessed based on youth practice of utilizing reproductive health services in the past one year, which includes family planning, sexually transmitted infections, diagnosis and treatment on sexual and reproductive related problems, voluntary counseling and testing, antenatal care, abortion care, post-abortion care, condom use, and general health information/counseling. Thus, using at least one component of the stated reproductive health services within the past one year was considered as the SRH service is utilized by the students.
- Sexual activity: Refers to the involvement in penetrative heterosexual intercourse within the last 12 months.
- Preparatory students: Those students who are in grades 11 and12.
- High school: A school, which entails grades 9 and 10
- Awareness about SRH Services: Those knowing the availability of one or more RH services were reported as having awareness about the services.
- Youth preferences: Preference of youth to health care workers and time for using SRH services.

Data Quality Control

A pretest was done on 5% of the sample size. The training was given for two consecutive days on how to administer questionnaires, handling ethical issues, and maintaining confidentiality and privacy. Completeness of each data collection tool had been checked by the principal investigator and the supervisors on a daily base. Double data entry was done by two data clerks and consistency of the entered data was cross-checked.

## Data Processing and Analysis

Data were entered, coded, cleaned, and checked by EpiData statistical software version 4.2.0.0, and analysis was done by using SPSS Version 20 statistical software. Binary logistic regression was used for analysis. The bivariable analysis was done and all variables that were found to be significant at p-value <0.25 were entered into the multivariable logistic regression model. Independent variables that were significant at p-value <0.05 in the multiple logistic regression models were considered as statistically significant. Finally, the data were presented in texts, figures, and tables.

## Ethical considerations

Ethical clearance was obtained from Gondar University, College of Medical and Health Sciences, Institutional Health Research Ethics Review Committee (IHRERC). Then Official letter had been written to each school for permission and support. A permission letter was obtained from the selected schools. The purpose of the study was explained to the study participants, informed written consent and assent were secured for children less than 18 years and confidentiality of the information was ensured. Data collected was used for the study purpose.

# Results

Socio-demographic characteristics of the respondents

In the study a total of 690 students were included, making a response rate of 99.1%. More than half, 376 (54.5%) were females. More than three fourth, 549 (79.6%) were within the age range of 15-19 years **(**Table 1**).**

Table 1: Socio-demographic characteristics students, Debre Tabor, Ethiopia, 2016

| Variables | Frequency | Percentage |
| --- | --- | --- |
| Sex |  |  |
| Male | 314 | 45.5 |
| Female | 376 | 54.5 |
| Age  15-19  20-24 | 549  141 | 79.6  20.4 |
| Ethnicity  Amhara  Others  Religion  Orthodox  Muslim  Others | 684  6  672  13  5 | 99.1  0.9  97.4  1.9  0.7 |
| Educational level |  |  |
| Grade- 9 | 115 | 16.7 |
| Grade-10 | 285 | 41.3 |
| Grade-11 | 144 | 20.9 |
| Grade-12  Residence  Rural  Urban | 146  372  318 | 21.2  53.9  46.1 |

Respondent’s family characteristics, living arrangement and sources of information

Four hundred and eighty-two (70.2%) of the respondent’s mothers had no formal education. More than half, 370 (53.6%) of the respondent’s fathers were farmers. One hundred eighty-two, (26.2%) of the respondents were living alone. Two-third, (73.2%) of the respondents had sources of information from radio and television **(**Table 2**).**

Table 2: Family characteristics, living arrangements, and communication means of students in Debre Tabor town, Northwest Ethiopia, 2016.

| Family characteristics | Frequency | Percentage |
| --- | --- | --- |
| Mother’s educational status |  |  |
| Unable to write and read  Able to write and read  Complete primary and secondary education  Diploma and above | 215  267  148  60 | 31.2  38.7  21.4  8.7 |
| Father’s educational status  Unable to write and read  Able to write and read  Complete primary and secondary education  Diploma and above  Mother’s occupational status  Housewife  M /trade vendor  Civil servant  Others*  Fathers occupational status  Farmer  Market trade vendor  Civil servant  NGO  Others** | 111  339  131  109  520  66  75  29  370  130  129  31  30 | 16.1  49.1  19.0  15.8  75.4  9.6  10.9  4.1  53.6  18.8  18.7  4.5  4.3 |
| Family alive  Mother  Father  Both alive  Both died  Respondent’s living arrangement | 69  23  596  2 | 10.0  3.3  86.4  0.3 |
| With both parents  Privately rented  Mother only  Father only  Living alone  With relatives  Others***  Communication means in the respondent’s house  Radio  Television  No communication means  Others**** | 364  182  59  15  34  51  19  213  292  174  11 | 52.8  26.2  8.6  2.2  4.9  7.4  2.8  30.9  42.3  25.2  1.6 |

*Note: * NGO, daily laborer, **daily laborers, car drivers, carpenters, *** with sister, brother, sexual partner; **** cell phone, newspaper, pamphlets*

Respondent’s awareness about reproductive health services

Six hundred and twenty (90%) of the respondents had awareness at least about one service, and 542 (78.6%) had information about the presence of an RH service facility in the town. However, only 30.9% of them had awareness of counseling services.

Youths’ preferences to time for seeking reproductive health services, to a type of health care providers, service fee and characteristics of health facilities

Two hundred and ninety-seven (43%) of the students preferred weekend time as the most favorable service hour and 286 (41.4%) preferred youth with the same sex as a health care provider. Three hundred and seventy-nine (54.9%) of the respondents described the health care providers as good and welcoming. However, 90 (13.0%) of the respondents reported that the health care providers were mistreated. 294 (42.6%) of students responded that the health care facility found in their respective residences was not suitable to use the services due to the mistreating health care providers, long-distance, and unfavorable service hours.

## Reproductive Health Services Utilization

Overall reproductive health utilization was 28.8%) [95% CI: 25.5-32.5]. Family planning, voluntary counseling, and testing for HIV, STI diagnosis and treatment, and general counseling services were utilized by 14.9%, 10.5%, 10.3%, and 7.9% youths respectively (Figure 2).

## Factors associated with reproductive health service utilization

In bivariate analysis, participant’s sex, residence, awareness of RH facility, awareness of RH service, discussion on RH services, ever had a sexual partner and penetrative sexual intercourse within the last year was identified as significant predictors of reproductive health service utilization.

However, multivariable logistic regression showed three variables, i.e being male, penetrative sexual intercourse within the last one year and adolescents who discussed reproductive health issues were associated with reproductive health service utilization.

Males were about 1.5 times more likely to utilize reproductive health services as compared to females (AOR = 1.54, 95% CI: 1.05, 2.25). Adolescents who discussed reproductive health issues were about 6.33 times more likely to use reproductive health services than those who had not discussed with health care workers, family, teachers, peers, and sexual partners(AOR = 6.33, 95% CI: 4.22, 9.51). Besides, adolescents who had penetrative heterosexual intercourse within the past one year were also nearly 2 times more likely to use the services than their counterparts(AOR =1.95, 95% CI: 1.10, 3.44)(Table 3).

Table 3: Factors associated with reproductive health service utilization among high school and preparatory students in Debre Tabor town, Northwest Ethiopia, 2016.

| Variables | Reproductive Health services utilization  Yes No | Crude OR (95% CI) | | AOR (95% CI) |
| --- | --- | --- | --- | --- |
| Sex |  | |  |  |
| Male  Female  Age  15-19  20-24  Residence  Rural  Urban  Participant’s educational level  High school  Preparatory  Respondent’s awareness of RH facility  Yes  No  Respondent’s awareness of RH services  Yes  No  Respondent’s discussion of RH service  Yes  No | 106 208  93 283  157 392  42 99  122 250  77 241  125 275  74 216  173 369  26 148  187 433  12 58  159 173  40 318 | | 1.56(1.11-2.16)  1.00  0.94(0.63-1.42)  1.00  1.53(1.09-2.14)  1.00    1.33(0.95-1.86)  1.00      2.20(1.39-3.49)  1.00    2.09(1.20-3.98)  1.00  7.30(4.93- 10.80)  1.00 | 1.54(1.05-2.25) *  1.00    0.94(0.59-1.52)  1.00  1.40(0.91-2.08)  1.00    1.30(0.93-2.03)  1.00    1.70 (0.99-2.92)  1.00  0.87(0.41-1.84)  1.00  6.33(4.22-9.51) **  1.00 |
| Ever had a sexual partner  Yes  No  Sexual intercourse within the last one year.  Yes  No | 66 97  133 394  46 46  153 445 | | 2.02(1.40-2.92)  1.00  2.91(1.86-4.55)  1.00 | 1.36(0.84-2.19)  1.00  1.95(1.10-3.44) *  1.00 |

Note: * significant at a p-value of < 0.05, ** significant at a p-value of < 0.01

# Discussion

The study revealed that the overall utilization of reproductive health services by high school and preparatory school students was found to be 28.8%, which was consistent with studies conducted in Badewacho woreda, southwest Ethiopia (29.4%) [18] and in Bahir Dar (32%) [19]. However, the findings of the present study are lower than the studies carried out in Dejen district, east Gojjam, Ethiopia,(45%) [20], in Jimma (34.7%) [21], and Harar, Ethiopia, (63.8%) [22]. The possible explanations for the difference of the study in Harar could be justified as; a higher proportion of married respondents (16.3%) in Harar may result in a higher proportion of service utilization. Furthermore, lower as compared to the studies done in Botswana [23] and England [24]. This might be due to differences in “the availability and accessibility of youth-friendly health facilities, youth centers, educational status, socioeconomic status, type of residence, transportation and culture.”

In this study sex, partner discussion about reproductive health issues, and sexual intercourse were significantly associated with RH service utilization. The study revealed that males were more likely to utilize reproductive health services as compared to females. This finding conforms with the study conducted in Nepal [25] and in Addis Ababa[9]. Nonetheless, it is different from the finding in Badewacho woreda, Hadiya zone, Ethiopia [18]. This might be due to cultural influences in the study area in which females have been still not allowed to go to health facilities for reproductive health services. It might also be because the proportion of male and female participants in our study was different from Badewacho woreda’s (Male: Female, 50.9:49.1).

This study also indicated that youth who had discussions about RH services with health workers, family, teachers, peers, and sexual partners were more likely to utilize the services than their counterparts. The finding of this study is consistent with the findings in Gondar [26] and Madawalabo [27]. This might be due to prior information on RH service increase their utilization, which would, in turn, motivate them to use the service. It might be also due to the possibility that youth who use services are also more likely to discuss services.

Students who had sexual intercourse within the past year were more likely to utilize RH service than abstainers. This finding is similar to the studies carried out in Gondar [26] and West Badewacho woreda, Ethiopia[18]. This might be because many of reproductive health service components (contraception) might be used when youths have perceived reproductive health risks related to sexual intercourse.

This study also tried to highlight that being too young to go for the services and inconvenient service hours were the two main reasons for them not to use sexual and reproductive health services for the students. This is similar to the study done in Bahir Dar, in Addis Ababa and Hara [28-30]. This might probably be due to the reason that students spent their time at school during the regular health institutions’ working hours and the institution may not be possibly functional at the weekend at which the students are relatively free. Besides, since the society declares the students as they are too young to go to the health institution due to some cultural influences and visiting health institutions for particular SRH services might be thought of as shameful.

Furthermore, it is found that lack of knowledge on the advantages of the services, long queue, inconvenient location of health facilities, lack of money, and the mistreating behavior of health care providers was found to be important barriers for youths to utilize sexual and reproductive health services. This is in line with the finding in Malawi and Kenya [31, 32]. This might be explained by the fact that most of the students may have little or no awareness about most of the sexual and reproductive health services are being offered free of charge. Besides, the students may also perceive that long queue may let them for unnecessary exposure to the peoples who might be around the health institution and this exposure might leave frustration related to their privacy and confidentiality. Hence, the situation might be exacerbated if they think that the location of the health institution is inconvenient for them. Therefore, educating female students to go to health facilities for SRH services, creating awareness on the preventive aspects of SRH problems, and advocating SRH service discussion among themselves and with others is important. Since the study is crossectional it does not show a cause-effect relationship. Those segments of the population who did not enter the school were missed. Factors from the service providers’ perspective, structural barriers as well as the supply was missed.

# Conclusions

Reproductive health service utilization among high school and preparatory students in Debre Tabor was low. Youth who are male, prior discussion on RH services to health workers, family, teachers, peers, and sexual partners and having penetrative heterosexual intercourse within the past one year were found to have significantly associated with reproductive health service utilization.

List of Abbreviations

ANC: Antenatal Care, AOR: Adjusted Odds Ratio, CI: Confidence Interval, FMOH: Federal Ministry of Health, FP: Family Planning, HIV: Human Immune Virus, NGO: Non-Governmental Organizations, OR: Odds Ratio, RH: Reproductive Health, SPSS: Statistical package for social sciences, SRH: Sexual and Reproductive Health, VCT: Voluntary Counselling and Testing, WHO: World Health OrganizationDeclarations

## *Ethical approval and consent to participate*: Ethical clearance was obtained from Gondar University, College of medical and Health Sciences, and Institutional Health Research Ethics Review Committee (IHRERC).

*Consent to publication*: Not applicable

*Availability of data and materials*: Data will be available upon request from the corresponding author and all relevant data are within the manuscript.

*Competing interests*: The authors declare that they have no competing interests.

*Funding*: Not applicable

*Authors’ Contribution: AS*, TA, MD, AA worked on designing the study, training the data collectors, supervising the data collectors, interpreting the result. AS, BM, YA Analyzed the result and wrote the manuscript. All authors involved starting from design, data interpretation, and critically review the manuscript.

# Acknowledgments: The authors acknowledged students who participate in the study and data collectors.

# References

1. Bernstein S, Hansen CJ: Public choices, private decisions: sexual and reproductive health, and the Millennium Development Goals. In: *UN Millennium Project: 2006*: Citeseer; 2006.

2. Blum RW, Mmari KN, Organization WH: Risk and protective factors affecting adolescent reproductive health in developing countries. 2005.

3. Carlo WA, McClure EM, Chomba E, Chakraborty H, Hartwell T, Harris H, Lincetto O, Wright LL: Newborn care training of midwives and neonatal and perinatal mortality rates in a developing country. *Pediatrics* 2010, 126(5):e1064-e1071.

4. Saxenian H: HPV vaccine adoption in developing countries: Cost and financing issues. *International AIDS Vaccine Initiative (IAVI)* 2007.

5. Cortez MA, Kubota H, de Curtis M, Ikeda A, Dudek FE, Galanopoulou AS.

6. Murphy E, Rudy S, Steele C, Kilbourne-Brook M: Improving interactions with clients: a key to high-quality services. *Outlook* 1999, 17(2):1-8.

7. Brieger WR, Delano GE, Lane CG, Oladepo O, Oyediran KA: West African Youth Initiative: outcome of a reproductive health education program. *Journal of Adolescent Health* 2001, 29(6):436-446.

8. Arnett JJ: Emerging adulthood: A theory of development from the late teens through the twenties. *American psychologist* 2000, 55(5):469.

9. Tigist t: Assessment of reproductive health service utilization and associated factors among high school youths in Addis Ababa, Ethiopia, 2015. Aau.

10. ICF CSACEa: Ethiopia Demographic and Health Survey 2016: Key indicators report. 2016.

11. Sawyer SM, Afifi RA, Bearinger LH, Blakemore S-J, Dick B, Ezeh AC, Patton GC: Adolescence: a foundation for future health. *The Lancet*, 379(9826):1630-1640.

12. Roberts JA, Iyer KK, Finnigan S, Vanhatalo S, Breakspear M: Scale-Free Bursting in Human Cortex following Hypoxia at Birth. *J Neurosci* 2014, 34(19):6557-6572.

13. El Waly B, Macchi M, Cayre M, Durbec P: Oligodendrogenesis in the normal and pathological central nervous system. *Front Neurosci* 2014, 8.

14. Yohannes B, Gelibo T, Tarekegn M: Prevalence and associated factors of sexually transmitted infections among students of Wolaita Sodo University, Southern Ethiopia. *Int J Sci Technol Res* 2013, 2(2):86-94.

15. Gelaye AA, Taye KN, Mekonen T: Magnitude and risk factors of abortion among regular female students in Wolaita Sodo University, Ethiopia. *BMC women's health* 2014, 14(1):50.

16. Andescavage NN, DuPlessis A, Limperopoulos C: Advanced MR Imaging of the Placenta: Exploring the in utero placenta-brain connection. *Epilepsia Open* 2015, 39(2):113-123.

17. Macro O, Measure D: Ethiopia Demographic and Health Survey, 2011: Preliminary Report: Central Statistical Agency.

18. Franceschini MA, Rosenthal ES, Chen X, Threlkeld SW, Cummings EE, Juan I, Makeyev O, Besio WG, Gaitanis J, Banks WA, *et al*: Ischemia-reperfusion impairs blood-brain barrier function and alters tight junction protein expression in the ovine fetus. *Neurophotonics* 2012, 226:89-100.

19. Abebe M, Awoke W: Utilization of Youth Reproductive Health Services and Associated Factors among High School Students in Bahir Dar, Amhara Regional State, Ethiopia. *Open Journal of Epidemiology* 2014, 2014.

20. Dagnew T, Tessema F, Hiko D: Health service utilization and reported satisfaction among adolescents in Dejen District, Ethiopia: A cross-sectional study. *Ethiopian Journal of health sciences* 2015, 25(1):17-28.

21. Tegegn A, Yazachew M, Gelaw Y: Reproductive health knowledge and attitude among adolescents: a community-based study in Jimma Town, Southwest Ethiopia. *Ethiopian Journal of Health Development* 2008, 22(3):143-151.

22. Motuma A: Youth-friendly health services utilization and factors in Harar, Ethiopia. *Harar Bulletin of Health Sciences* 2012, 4:15-21.

23. Wittet S, Tsu V: Cervical cancer prevention and the Millennium Development Goals. *Bulletin of the World Health Organization* 2008, 86(6):488-490.

24. Waiswa P, Peterson S, Tomson G, Pariyo GW: Poor newborn care practices-a population-based survey in eastern Uganda. *BMC pregnancy and childbirth* 2010, 10(1):9.

26. Kim J, Choi IY, Dong Y, Wang WT, Brooks WM, Weiner CP, Lee P: Chronic fetal hypoxia affects axonal maturation in guinea pigs during development: a longitudinal Diffusion Tensor Imaging and T2 mapping study. *J Magn Reson Imaging* 2015, 42(3):658-665.

27. 91st Annual Conference of the Canadian Paediatric Society: Abstract Titles: Abstracts to be delivered at the 91st Annual Conference of the Canadian Paediatric Society, Montreal, Quebec, June 25 to 28, 2014, alphabetized according to the first author’s surname. The Canadian Paediatric Society website provides scheduling information: www.annualconference.cps.ca/ resources/pro gram Convention registrants will receive a complete program on-site. Full-text abstracts appear on the Paediatrics & Child Health website. *Paediatr Child Health* 2014, 19(6):293-298.

28. Carlo WA, Goudar SS, Jehan I, Chomba E, Tshefu A, Garces A, Sailajanandan P, Althabe F, McClure EM, Derman RJ: Newborn-care training and perinatal mortality in developing countries. *New England Journal of Medicine* 2010, 362(7):614-623.

29. Grady K, Ameh C, Adegoke A, Kongnyuy E, Dornan J, Falconer T, Islam M, Van Den Broek N: Improving essential obstetric and newborn care in resource-poor countries. *Journal of Obstetrics and Gynaecology* 2011, 31(1):18-23.

30. Chomba E, McClure EM, Wright LL, Carlo WA, Chakraborty H, Harris H: Effect of WHO newborn care training on neonatal mortality by education. *Ambulatory Pediatrics* 2008, 8(5):300-304.

31. Castro KG, Ward JW, Slutsker L, Buehler JW, Jaffe HW, Berkelman RL, Curran JW: 1993 revised classification system for HIV infection and expanded surveillance case definition for AIDS among adolescents and adults. *MMWR Recomm Rep* 1992, 41(RR-17):1-19.

32. Baqui AH, El-Arifeen S, Darmstadt GL, Ahmed S, Williams EK, Seraji HR, Mannan I, Rahman SM, Shah R, Saha SK: Effect of community-based newborn-care intervention package implemented through two service-delivery strategies in Sylhet district, Bangladesh: a cluster-randomized controlled trial. *The lancet* 2008, 371(9628):1936-1944.

# List of figures

Figure 1: Schematic presentation of sampling procedures

Figure 2: Components of reproductive health services mentioned by high school and preparatory school students, Debre Tabor, Ethiopia, 2016
