# Supplementary material for: Youth friendly sexual and reproductive health service utilization among high and preparatory school students in Debre Tabor town, Northwest Ethiopia: A cross sectional study
Source: PLoS One. 2020 Sep 30;15(9):e0240033. doi: 10.1371/journal.pone.0240033 (PMC7526918; doi:10.1371/journal.pone.0240033)
Supplement: Attachment — Submitted filename: Response to reviewers.docx [file pone.0240033.s009.docx]

Dear Editors and reviewer;

Thank you very much for your critical comments and suggestions. The comments are helpful in in the shaping of the manuscript. The suggestions and comments have been closely followed and revisions have been made accordingly. The following are the questions extracted from the reviewers’ comments along with our summarized responses.

Reviewer #1. Incomplete? Extensive language editing is important not only for this section but other sections of your manuscript. ?? As you know, in urban settings these subgroup of populations are in learning institutions and all that you have said above is doing for these group (schools are settings that are best to reach adolescents).

Author response: the abstract section has been edited and included in manuscript file. We tried to use online grammar checker.

Reviewer comment: Proper leveling of headings and subheadings of section of the manuscript may be important

Author response: heading and sub heading has been rearranged as heading one ,heading two, heading three
Reviewer comment: Sampling techniques need some revision as per comments provided .Read it what “PPS” IS really mean and revise accordingly ? Or” The calculated Sample size was proportionally allocated for selected schools” is enough for this study. In PPS, there are issues like cumulative proportion and probabilities for each corresponding cluster that is commonly suggested methods for large numbers of clusters.

Author response: The sentence “The number of respondents in the two schools (secondary and preparatory) was determined using probability proportionate to size allocation method” is replaced by “the calculated Sample size was proportionally allocated for selected schools. The sampling techniques has be revised as per the comment given.

Reviewer comment: Check epidata version 4.2.0.0

Author response: we have checked and it is epilate 4.2.0.0

Reviewer #3: The term ' sexual and reproductive' should be added to the title to reflect the content of the manuscript. I suggest 'Youth friendly sexual and reproductive health service utilization among high and preparatory school students in Debre Tabor Town, Northwest Ethiopia: a cross sectional study.

Author response: based on the comment given the title has been corrected as “Youth friendly sexual and reproductive health service utilization among high and preparatory school students in Debre Tabor Town, Northwest Ethiopia: a cross sectional study
Reviewer comment: Sample size calculation. This should be written in one paragraph. The formula is not necessary.

Author response: the formula was omitted as per the comment and summarized in one paragraph

Reviewer comment: The variables could be defined under the variables section.
Author response: really we can place under variable section, we make it under operational section to make easier for reader.

Reviewer comment: Ethical clearance was obtained from Gondar University, College of Medical and Health Sciences, Institutional Health Research Ethics Review Committee (IHRERC).Please provide the ethics approval/ registration number.

Author response: ethics approval/registration number was: Ref no.CMHS/415/2016

Reviewer comment: Was parental consent taken for those below 18 years old? If yes, this needs to be written in the manuscript.

Author response: Yes, it was included as “For children less than 18 years assent (written consent) were secured from their guardians/parents and confidentiality of the information was ensured”

Reviewer comment :294 (42.6%) of students responded that the health care facility found in their respective residences was not suitable to use the services due to the mistreating health care providers, long-distance, and unfavorable service hours.
Reviewer: Avoid begin a sentence with a number.
Author response: it is corrected as two hundred ninety four, 42.6% of students responded that the health care facility found in their respective residences was not suitable to use the services due to the mistreating by health care provider

Reviewer comment: However, 90 (13.0%) of the respondents reported that the health care providers were mistreated. Shouldn’t this be the other way round? The youths were mistreated instead of the healthcare providers.

Author response: corrected as “However, 90(13.0%) of youth were mistreated by health care providers.

Reviewer comment: This might be due to differences in “the availability and accessibility of youth-friendly health facilities, youth centers, educational status, and socioeconomic status, type of residence, transportation and culture.” Is this an assumption or evidence-based?

Author response; this is an assumption
This might be due to cultural influences in the study area in which females have been still not allowed to go to health facilities for reproductive health services. Provide evidence for this statement.

Author response: corrected as “This might be due to cultural influences in the study area in which females was less empowered. (References cited on the main document)

Reviewer comment: Authors should add future direction of the research based on study limitations.

Author response: based on this study limitation we recommend the following. So future research should be done on effect of poor utilization of reproductive health services on health outcome by including variables such as service providers’ perspective and structural barriers.
